# Pancytopenia, Cholestatic Jaundice, and Pulmonary Hypertension: Rare Extrathyroidal Manifestations of Graves’ Disease

**DOI:** 10.7759/cureus.71175

**Published:** 2024-10-09

**Authors:** John Khor, Teoh Jamie Hong Im

**Affiliations:** 1 Internal Medicine, Hospital Sultanah Nora Ismail, Batu Pahat, MYS; 2 Endocrinology, Hospital Sultanah Aminah, Johor Bahru, MYS

**Keywords:** antithyroid drug, cardiomyopathy, cholestatic jaundice, graves’ disease, pancytopenia

## Abstract

Pancytopenia, cholestatic jaundice, and pulmonary hypertension are rare but serious complications of thyrotoxicosis. It is uncommonly seen in patients with hyperthyroidism and may result in treatment-related dilemmas as bone marrow suppression and drug-induced liver injury are known complications of anti-thyroid treatment.

We describe a case of Graves’ disease presenting with all three manifestations of pancytopenia, cholestatic jaundice, and pulmonary hypertension during a relapse of thyrotoxicosis. Her anti-thyroid medications were initially withheld for fear of worsening the above manifestations, but no improvement was noted clinically or biochemically. Other lab investigations, such as hepatitis serology, autoimmune liver markers, and ultrasound, were unrevealing. Anti-thyroid medications were subsequently reinitiated after discussion with the endocrine team, with improvements in clinical condition, as well as full blood counts and liver function tests. Pulmonary hypertension also improved on her latest echocardiography.

This case report intends to shed light on the atypical extrathyroidal manifestations of Grave's disease and the importance of recognition and therapy.

## Introduction

Diffuse goiter, orbitopathy, pretibial myxedema (thyroid dermatopathy), and thyroid acropachy are pathognomonic features that define Grave's disease (GD); other extrathyroidal complications such as pancytopenia [1] and cholestatic jaundice [2] are rare. Only 30 cases of pancytopenia have been reported in association with GD while cholestatic jaundice is seen in only 12% of GD patients. In contrast, while GD-associated pulmonary hypertension is common (prevalence of 36-65%), patients are generally asymptomatic [3]. These three extrathyroidal manifestations have been reported in isolation and rarely together [1,4]. In this case report, we discuss a patient presenting with all three manifestations of pancytopenia, cholestatic jaundice, and pulmonary hypertension during a relapse of thyrotoxicosis.

## Case presentation

A 52-year-old housewife with no known illness was diagnosed with GD two years ago and had been on carbimazole treatment with labile thyroid function tests (TFTs). She developed pancytopenia, cholestatic jaundice, and atrial fibrillation (AF) in July 2023 when she experienced a relapse of thyrotoxicosis (Table 1).

**Table 1:** Serial laboratory investigations fT4: free T4; TSH: thyroid-stimulating hormone; Hb: hemoglobin; WBC: white blood count; Plt: Platelets; ANC: absolute neutrophil count; ALT: alanine transaminase; ALP: alkaline phosphatase; CMZ: carbimazole; PTU: propylthiouracil

Lab test	Normal range	Units	Feb 23	March 23	May 23	July 23	Aug 23	Oct 23	Nov 23	Dec 23	Feb 24	March 24	May 24
TSH	0.27-4.20	mIU/L	<0.0083	<0.0083	<0.0083	<0.0083	<0.0083	<0.0083	<0.0083	<0.0083	<0.0083	<0.0083	1.61
fT4	12-22	pmol/L	8.2	6.0	19	>64.35	46.2	20.5	<5.41	41.9	64.35	6.7	< 5.41
Hb	12 – 14	g/dL	13.4	12.6	12.3	10	9.5	10.7	-	9.9	8.9	10.8	12
WBC	4-10	10^3/uL	4.88	4.54	5.23	6.1	3.11	3.62	-	2.81	3.57	4.13	4.7
Plt	150 – 400	10^3/uL	134	166	142	97	30	132	-	80	74	134	156
ANC	2.0-7.0	10^3/uL	1.8	0.6	1.1	2.4	1.2	1.1	-	1.2	1.0	1.49	1.98
Total protein	60 – 80	g/L	77	-	76	66	-	72	71	70	70	75	83
ALT	25 – 35	U/L	9	-	12	17	-	9	7	16	18	10	11
Bilirubin	5 – 20	µmol/L	19.5	-	28	68.6	-	47.9	32.2	100.7	104.9	33.7	19.2
ALP	35 – 130	U/L	110	-	134	147	-	240	265	178	184	200	149
Medication			CMZ	CMZ	CMZ	CMZ	PTU	PTU	Stop	PTU	PTU	CMZ	CMZ
Dose			5 mg OD	10 mg OD	10 mg OD	10 mg OD	100 mg TDS	100 mg TDS	Stop	100 mg BD	100 mg BD	40 mg OD	40 mg OD

She complained of heat intolerance and inability to gain weight despite a good appetite. For fear of worsening pancytopenia and cholestatic jaundice, the antithyroid drug (ATD) was switched from carbimazole to propylthiouracil. This led to a fluctuation in TFTs, persistent pancytopenia, and cholestatic jaundice (Table 1).

**Figure 1:**
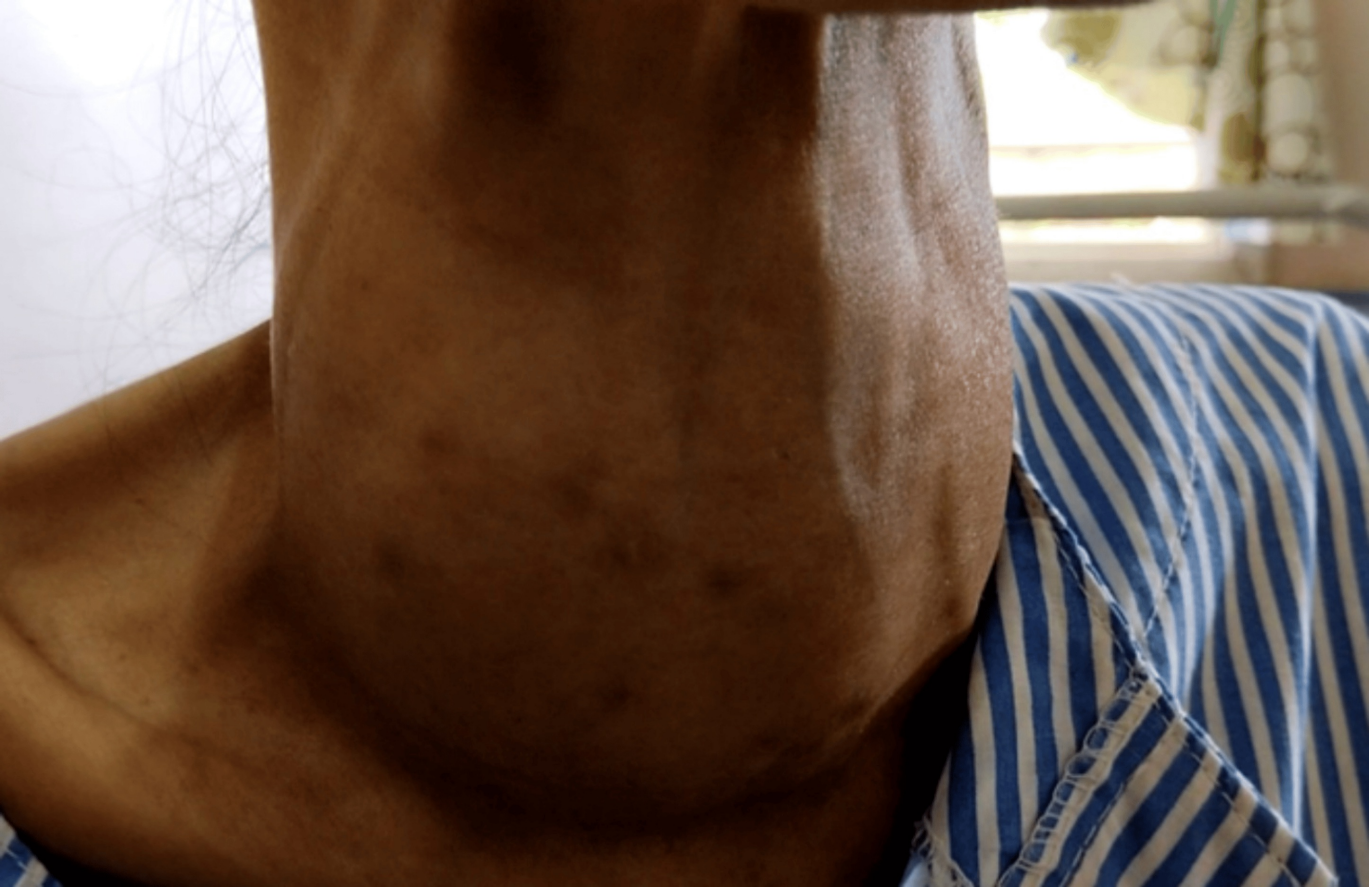
Thyroid Goiter

**Figure 2:**
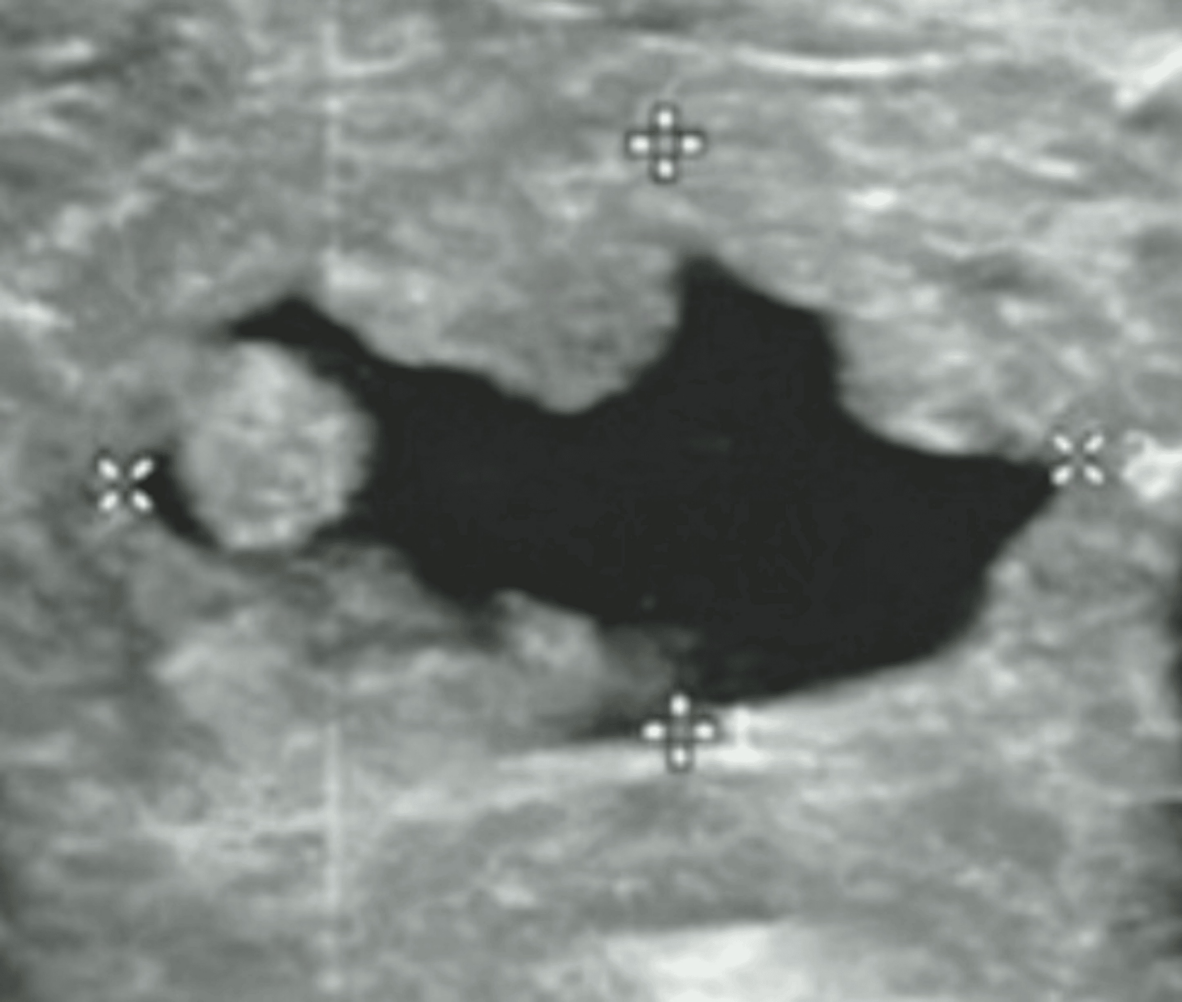
Nodule a: well-defined mixed solid cystic lesion in right thyroid lobe measuring 0.9 x 1.5 x 1.7 cm

**Figure 3:**
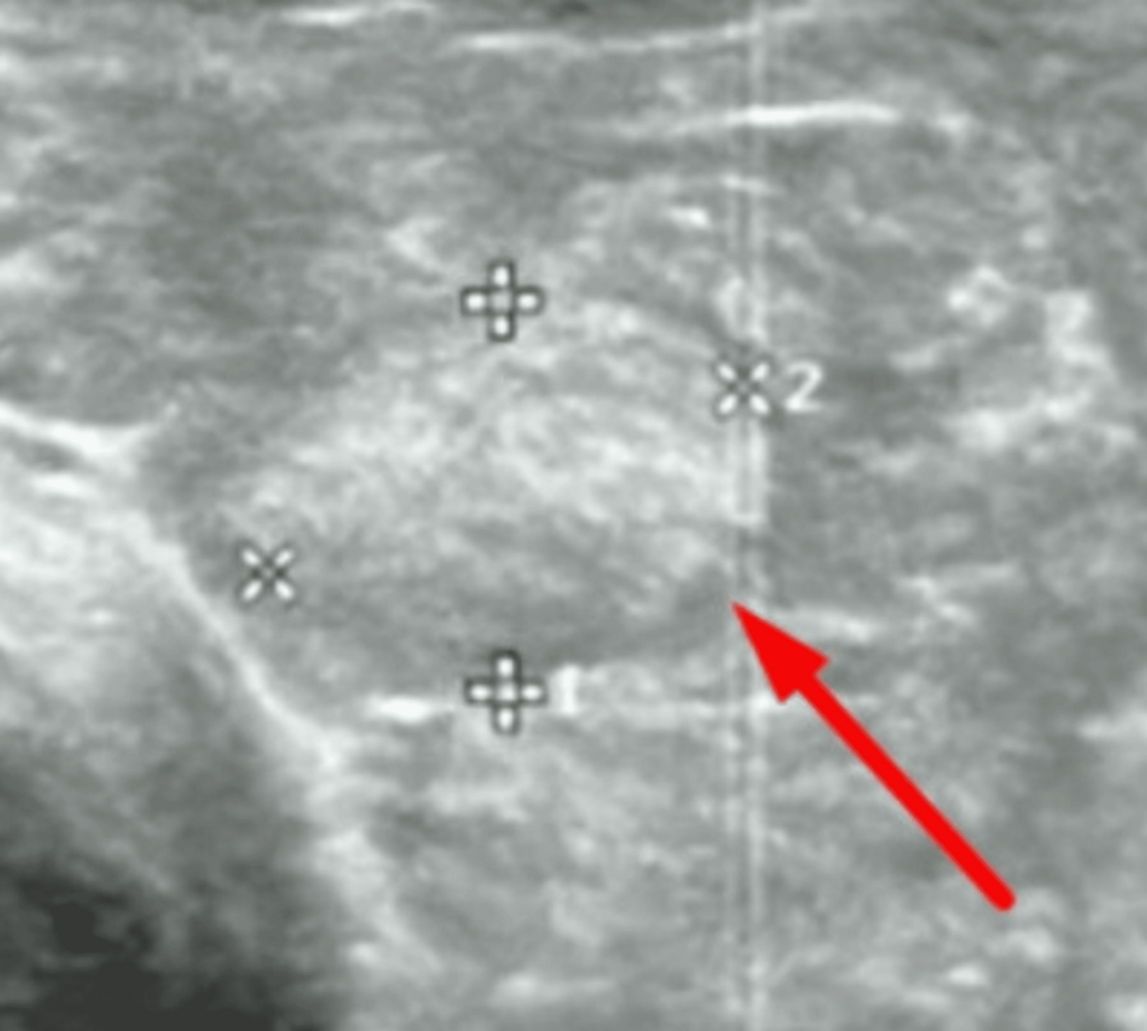
Nodule b: isoechoic nodule seen at left thyroid lobe measuring 0.6 x 0.8 x 0.6 cm with no vascularity or calcification

She was otherwise emaciated, with a BMI of 14.3 kg/m^2^. Bilateral exophthalmos, a huge diffuse goiter with a loud thyroid bruit was appreciated (Figure 1). Conjunctival pallor and sclera icterus were also seen in her eyes while a loud second heart sound, deviated apex beat, and pulsatile liver were also present. An echocardiogram (ECHO) revealed severe tricuspid regurgitation (TR) of the peak pressure gradient (PPG) of 45 mmHg with pulmonary artery systolic pressure (PASP) of 60 mmHg, suggestive of severe pulmonary hypertension (PHT). Left ventricular function was otherwise preserved (ejection fraction of 50-55%), while all heart chambers (right atrium (RA), right ventricle (RV), and left atrium (LA)) were dilated. Ultrasonography (US) of the thyroid revealed a diffuse heterogeneous echotexture with increased vascularity in both lobes suggestive of autoimmune thyroiditis, with two nodules (nodule a: Figure 2 and nodule b: Figure 3) noted**. **Anti-TSH antibody (TRAb) was raised at 39 IU/L (< 1.75). Peripheral blood film, the Coombs test, tests for rheumatological diseases and viral and autoimmune hepatitis (antimitochondrial antibodies (AMA), anti-smooth muscle antibodies (ASMA), anti-liver-kidney microsomal (anti-LKM) antibodies), and US hepatobiliary system (HBS) were unrevealing.

**Figure 4:**
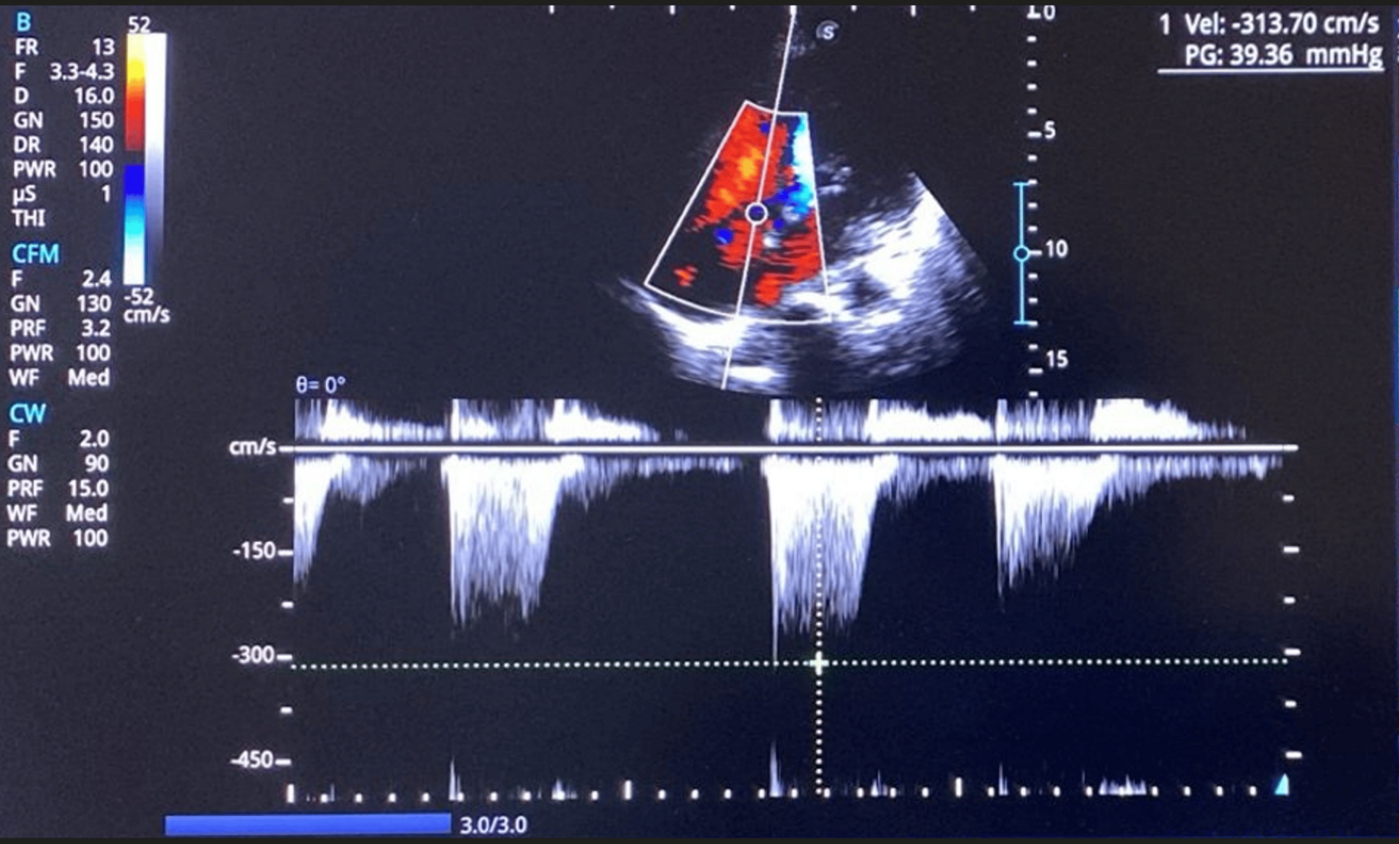
Echocardiography post-treatment (peak pressure gradient improved to 39 mmHg)

After an endocrine consultation in February 2024, carbimazole 40 mg once a day (OD) was started. Six weeks later, the TFTs, full blood count (FBC), and liver function tests (LFTs) were substantially improved (Table 1). Her ECHO also depicted improvement of PHT with PASP 47 mmHg and moderate TR (PPG 39 mmHg) (Figure 4).

Carbimazole was tapered down to 10 mg OD and she underwent thyroidectomy successfully. Post-operatively, she was initiated on levothyroxine 100 mcg OD. To date, she is euthyroid with a normal FBC and normal LFTs.

## Discussion

In our patient, a biochemical improvement of pancytopenia and cholestatic jaundice was noted from October to November 2023 (Table 1). However, this was not recognized, and the ATD was withheld for fear of overtreatment. This led to rebound thyrotoxicosis and worsening of pancytopenia and cholestatic jaundice in December 2023. The biochemical improvement after attaining euthyroidism suggests thyrotoxicosis as the cause of both manifestations, as opposed to ATD [1-3]. 

Graves’ pancytopenias are rarely reported. Ineffective hematopoiesis, reduction in erythrocyte life span due to hypersplenism, autoimmune antibodies towards erythrocyte, or even toxicity of thyroid hormone to bone marrow are proposed mechanisms [1,3]. Moreover, GD can coexist with other autoimmune syndromes, such as pernicious anemia, aplastic anemia, or autoimmune hepatitis, which must be excluded. Understandably, ATDs can also cause hepatitis, agranulocytosis, or rarely pancytopenia [1]; severity can be dose-dependent [5].

Liver injury in GD can take many forms, from elevated alkaline phosphatase (ALP, 33%), alanine transaminase (ALT, 26%), gamma-glutamyl transferase (GGT, 24%), aspartate aminotransferase (AST, 17%), and hyperbilirubinemia (8%) [4]. The last manifested as cholestatic jaundice in our patient. Co-existing viral, alcoholic, and drug-induced hepatitis are possibilities that must be first excluded [2]. In clinically hyperthyroid patients, the mismatch between splanchnic blood supply and the demand for hepatocytes with increased metabolic activity, direct toxicity of thyroid hormones, or congestive heart failure (CCF) are postulated pathophysiological mechanisms [4]. On a cellular level, this is attributed to triiodothyronine (T3)-mediated apoptosis of hepatocytes via fatty infiltration, nuclear irregularity, nuclear hyperchromatism, and cytoplasm vacuolization [2].

The cardinal features of thyrotoxic cardiomyopathy are left ventricular hypertrophy, rhythm disturbances, dilatation of heart chambers, CCF, PHT, and diastolic dysfunction [4]. T3 is associated with increased diastolic function, inotropy, and chronotropy [6]. The ensuing high cardiac output, coupled with endothelial injury and increased metabolisms of pulmonary vasodilators, are thought to mediate PHT, while shortened refractory periods are thought to result in AF [3]. Reversibility or improvement of both has been reported after ATD initiation or thyroidectomy due to the normalization of pulmonary blood flow on euthyroidism [3,6]. Our patient has atrial fibrillation, PHT, and dilated chambers. Right heart failure manifested as PHT and TR in our patient may also result in liver congestion and hyperbilirubinemia. Nevertheless, the PHT and TR improved after thyroidectomy, with normalization of RV enlargement; however, the AF persists.

After excluding other pathologies for pancytopenia and cholestatic jaundice, antithyroid therapy was re-initiated. The clinical improvement confirmed both pancytopenia and cholestatic jaundice were caused by thyrotoxicosis. Ultimately, given the large goiter, the patient required thyroidectomy as the definitive therapy of GD.

## Conclusions

Graves' hyperthyroidism may manifest as pancytopenia, cholestatic jaundice, or pulmonary hypertension. These manifestations might co-occur, as seen in this case report. They must be considered in overt hyperthyroid patients, especially if local, drug, autoimmune, and infective causes have been ruled out. Anti-thyroid medications should be intensified and the patient should be monitored accordingly. A successful therapeutic trial is confirmatory. Treatment is important to prevent other life-threatening complications. 
